# MRI detection of the malignant transformation of stem cells through reporter gene expression driven by a tumor-specific promoter

**DOI:** 10.1186/s13287-021-02359-w

**Published:** 2021-05-12

**Authors:** Jun Sun, Jie Huang, Guangcheng Bao, Helin Zheng, Cui Wang, Jie Wei, Yuanqiao Fu, Jiawen Qiu, Yifan Liao, Jinhua Cai

**Affiliations:** 1 Department of Radiology, Children’s Hospital of Chongqing Medical University, Chongqing, 400014 China; 2 Ministry of Education Key Laboratory of Child Development and Disorders, Chongqing, 400014 China; 3 Key Laboratory of Pediatrics in Chongqing, Chongqing, 400014 China; 4 Chongqing International Science and Technology Cooperation Center for Child Development and Disorders, Chongqing, 400014 China; 5 Department of Radiology, Chongqing University Central Hospital, Chongqing, 400014 China; 6 Department of Nuclear Medicine, Xinqiao Hospital affiliated with Third Military Medical University, Chongqing, 400037 China

**Keywords:** Promoter of progression elevated gene-3, Ferritin heavy chain, Magnetic resonance imaging, Stem cells, Malignant transformation

## Abstract

**Background:**

Existing evidence has shown that mesenchymal stem cells (MSCs) can undergo malignant transformation, which is a serious limitation of MSC-based therapies. Therefore, it is necessary to monitor malignant transformation of MSCs via a noninvasive imaging method. Although reporter gene-based magnetic resonance imaging (MRI) has been successfully applied to longitudinally monitor MSCs, this technique cannot distinguish the cells before and after malignant transformation. Herein, we investigated the feasibility of using a tumor-specific promoter to drive reporter gene expression for MRI detection of the malignant transformation of MSCs.

**Methods:**

The reporter gene ferritin heavy chain (FTH1) was modified by adding a promoter from the tumor-specific gene progression elevated gene-3 (PEG3) and transduced into MSCs to obtain MSCs-PEG3-FTH1. Cells were induced to undergo malignant transformation via indirect coculture with C6 glioma cells, and these transformed cells were named MTMSCs-PEG3-FTH1. Western blot analysis of FTH1 expression, Prussian blue staining and transmission electron microscopy (TEM) to detect intracellular iron, and MRI to detect signal changes were performed before and after malignant transformation. Then, the cells before and after malignant transformation were inoculated subcutaneously into nude mice, and MRI was performed to observe the signal changes in the xenografts.

**Results:**

After induction of malignant transformation, MTMSCs demonstrated tumor-like features in morphology, proliferation, migration, and invasion. FTH1 expression was significantly increased in MTMSCs-PEG3-FTH1 compared with MSCs-PEG3-FTH1. Prussian blue staining and TEM showed a large amount of iron particles in MTMSCs-PEG3-FTH1 but a minimal amount in MSCs-PEG3-FTH1. MRI demonstrated that the T2 value was significantly decreased in MTMSCs-PEG3-FTH1 compared with MSCs-PEG3-FTH1. In vivo, mass formation was observed in the MTMSCs-PEG3-FTH1 group but not the MSCs-PEG3-FTH1 group. T2-weighted MRI showed a significant signal decrease, which was correlated with iron accumulation in the tissue mass.

**Conclusions:**

We developed a novel MRI model based on FTH1 reporter gene expression driven by the tumor-specific PEG3 promoter. This approach could be applied to sensitively detect the occurrence of MSC malignant transformation.

## Background

In recent years, mesenchymal stem cells (MSCs) have attracted increasing interest in tissue engineering and cell-based therapy owing to their self-renewal ability, multipotent differentiation potential, and paracrine effects [1–5]. However, existing evidence shows that MSCs can undergo malignant transformation, although the exact underlying mechanism remains unclear [6–10]. This has made the safety of MSC-based therapeutic strategies questionable and seriously impeded their application. Therefore, it is especially important to detect tumor formation as early as possible by tracing transplanted stem cells in vivo.

At present, optical, nuclear medicine, and magnetic resonance imaging (MRI) have been used in the field of stem cell tracing in vivo [11, 12]. Among these imaging modalities, MRI has the most potential due to its advantages, including nonionizing radiation, high spatial resolution, and deep tissue penetration [13, 14]. MRI tracing of stem cells includes two methods: direct imaging by labeling cells with magnetic nanoparticles in advance and indirect imaging by using an MRI reporter gene. For the former method, intracellular magnetic nanoparticles can diminish with cell division, which limits the potential of this approach for long-term monitoring [15–17]. For the latter method, an MRI reporter gene can be expressed persistently and recruit iron particles into cells, which enables longitudinal monitoring of the labeled cells [18–21].

In previous studies, we used ferritin heavy chain (FTH1) as an MRI reporter gene to detect transplanted MSCs in vivo and confirmed the feasibility of tracing cells in a sustained mode [18, 20]. However, this reporter gene-based monitoring strategy could not be used to distinguish malignantly transformed cells from the originally transplanted stem cells. The key to solving this problem is to find a gene that is differentially expressed before and after stem cell malignant transformation, that is, a gene highly expressed in tumorigenic cells but not in stem cells or normally differentiated tissue cells. By using such a differently expressed gene promoter to drive the specific expression of the reporter gene in tumorigenic cells, it would be possible to detect the occurrence of malignant stem cell transformation.

Progression elevated gene-3 (PEG3) is a tumor-specific gene derived from rodents. Studies have revealed that PEG3 promoter activity is regulated by two transcription factors, PEA3 and AP-1, which are highly expressed in human cancer cell lines but rarely expressed in normal tissue cells [22, 23]. At present, the PEG3 promoter has been applied in the research field of targeted cancer therapy and investigation of the mechanism of tumorigenesis [24–28]. However, MRI monitoring of the tumorigenicity of stem cells by using tumor-specific promoter-triggered expression of a reporter gene has not been reported.

**Fig. 1:**
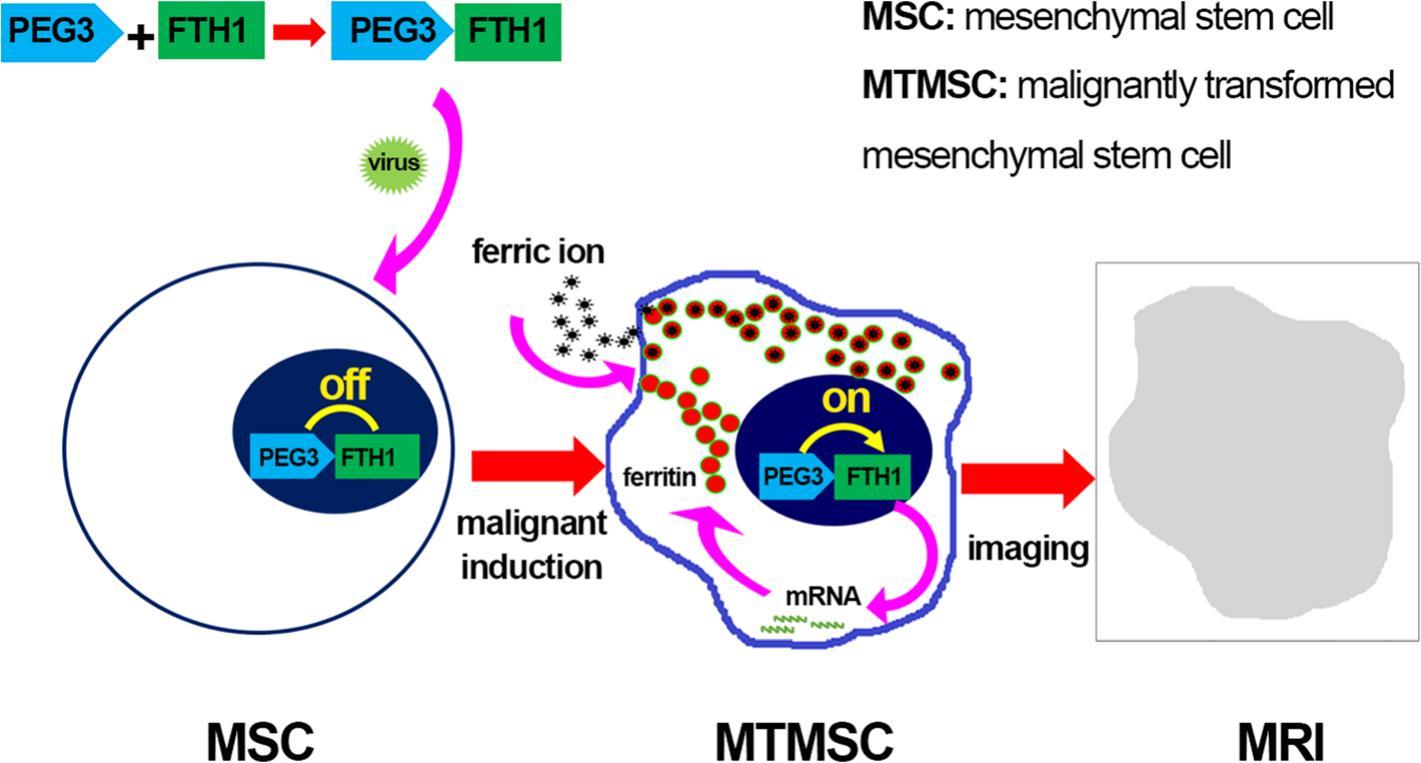
Cellular MRI based on FTH1 expression driven by the tumor-specific PEG3 promoter

In this study, we combined the promoter PEG3 and MRI reporter gene FTH1 to construct the lentiviral vector (LV) PEG3-FTH1-LV and transferred the PEG3-FTH1 system into bone marrow-derived MSCs. In the process of inducing malignant transformation of MSCs, reporter gene expression and the resulting MRI signal were observed and assessed (Fig. 1). The aim of the experiment was to explore the feasibility of using the tumor-specific promoter PEG3 to drive the expression of the reporter gene FTH1 for MRI detection of the malignant transformation of bone marrow-derived MSCs.

## Methods

### Ethics statement

All animal protocols used in this study were reviewed and approved by the Animal Care and Use Committee of Chongqing Medical University, and the experimental procedures were performed in accordance with the National Institutes of Health guidelines. All efforts were made to minimize animal suffering.

### Culture and identification of MSCs

Four-week-old Sprague-Dawley (SD) rats (40–60 g) from the Experimental Animal Center at Chongqing Medical University were used for isolation and culture of MSCs according to previous methods [29]. Briefly, the long bones of the four limbs of rats were isolated under pathogen-free conditions. The bone marrow cavity was washed with Dulbecco’s modified Eagle’s medium (DMEM; Gibco, Grand Island, NY, USA), and the cells were centrifuged, resuspended in a washing solution and then cultured in DMEM/F12 supplemented with 10% fetal bovine serum (FBS; HyClone, Logan, UT, USA), 100 units/ml penicillin, and 100 g/ml streptomycin at 37°C under 5% CO_2_. Third-generation cells with an excellent growth status were digested with 0.25% trypsin, collected, centrifuged at 4°C (1000 r/min, 5 min), washed with phosphate-buffered saline (PBS) thrice, and stored for future use.

To identify the phenotypic characteristics of stem cells, monoclonal antibodies specific for CD29, CD34, CD45, and CD90 (Beyotime, Nanjing, Jiangsu, China) were separately added to cells and incubated in the dark for 30 min. The cells were washed with PBS thrice and resuspended in 200 μl of PBS; they were then detected and analyzed using a flow cytometer (BD Biosciences, Franklin Lakes, NJ, USA).

### Construction of recombinant lentiviral vectors and production of viruses

The rat-derived PEG3 promoter (−101 to +190 bp) was amplified by polymerase chain reaction (PCR) using rat genomic DNA as the template with the following primer pair: forward, 5’-TCCGGTGAATTCGCCACCATGACGACCGCGT-3’; and reverse, 5’-GCAGATCCTTACTAGTATCGATGGATC-3’. The human FTH1 gene (accession number BC000857) was amplified by PCR with the following primer pair: forward, 5’-AACCGTCAGATCGCACCGGTGCCACCATGACGACCGCGTCCACCTC-3’; and reverse, 5’-TCCTTGTAGTCCATGAATTCGCTTTCATTATCACTGTCTC-3’. The PEG3 promoter and FTH1 were inserted into the lentiviral vector pHBLV-CMV-MCS-3flag-EF1-ZSgreen-puro by using two ClaI sites (2180 and 2942) to generate the recombinant lentiviral vector pHBLV-PEG3-FTH1-3flag-EF1-ZSgreen-puro (LV-PEG3-FTH1); FTH1 alone was inserted into the lentiviral vector pHBLV-CMV-MCS-3flag-EF1-ZSgreen-puro by using a Xba and BamHI double-digestion system to generate pHBLV-CMV-FTH1-3flag-EF1-ZSgreen-puro (LV-CMV-FTH1) as the positive control. FTH1 gene and PEG3 promoter cDNA sequences were verified by PCR and DNA sequencing. Plasmids of each group were validated by agarose gel electrophoresis and DNA sequencing. Lentiviruses were generated by cotransfecting LV-CMV-FTH1 or LV-PEG3-FTH1 together with pSPAX2 and pMD2G into 293T packaging cells (Invitrogen, Carlsbad, CA, USA). Fresh medium containing 10% FBS was added 6 h after transfection, and then, the viral medium was collected at 48 and 72 h. Cell debris was removed by centrifugation at 4°C and 2000×*g* for 10 min. Viruses were enriched by centrifugation at 4°C and 82,700×*g* for 120 min and stored at −80°C.

### Recombinant lentiviral transfection of MSCs

MSCs at 30–40% confluence were infected with lentiviruses using 5-μg/ml polybrene (Sigma-Aldrich, St. Louis, MO, USA). In the experimental and positive control groups, MSCs were infected with LV-PEG3-FTH1 or LV-CMV-FTH1, respectively, at a multiplicity of infection (MOI) of 5, 10, 15, 20, or 30. In the negative control group, MSCs were treated with an equal volume of PBS. The fresh medium containing 10% FBS was refreshed at 12 h. Green fluorescence was viewed with fluorescence microscopy to evaluate the expression of GFP at 24 h, 48 h, and 72 h after infection. The best MOI was determined from the fluorescence intensity and cell state results. The infected MSCs were cultured in medium supplemented with puromycin (Sigma-Aldrich, St. Louis, MO, USA) at a concentration of 0.1, 0.5, 1.0, 1, 2, 3, 4, or 5 μg/ml to identify the minimum lethal concentration at 7 days. The medium was supplemented with a minimum lethal concentration of puromycin for 7 days to generate clonal cell lines (MSCs-PEG3-FTH1 and MSCs-CMV-FTH1).

### Malignant transformation induction and assessment

MSCs, MSCs-PEG3-FTH1, and MSCs-CMV-FTH1 were induced to undergo malignant transformation by indirect coculturing with C6 glioma cells as described previously [30]. Briefly, rat C6 cells were cultured in DMEM/F12 supplemented with 10% FBS for 24 h, and then, the culture medium was collected and filtered through a 0.4-μm filter. The collected culture medium was added to fresh culture medium at a ratio of 1:1. The mixed medium was used to culture MSCs, MSCs-PEG3-FTH1, and MSCs-CMV-FTH1. The culture medium was changed every day. After 2 weeks of induction, the cells were collected and named MTMSCs, MTMSCs-PEG3-FTH1, and MSCs-CMV-FTH1, respectively.

To identify the phenotypic characteristics of the induced cells, the expression of the surface antigens CD34, CD45, CD29, and CD90 on MTMSCs, MTMSCs-PEG3-FTH1, and MTMSCs-CMV-FTH1 was detected by flow cytometry as described before.

Cell viability was assayed with Cell Counting Kit-8 (CCK-8; Dojindo Laboratories, Kumamoto, Japan) according to the manufacturer’s protocols. Cells were divided into 6 groups: the MSC, MSC-PEG3-FTH1, MSC-CMV-FTH1, MTMSC, MTMSC-PEG3-FTH1, and MTMSC-CMV-FTH1 groups. Each group of cells was seeded in 96-well plates (1 × 10^3^ cells/well, 6 wells per group). At 1 days, 2 days, 3 days, 4 days, 5 days, and 6 days after seeding, the cells were incubated with 100 μl of serum-free DMEM/F12 containing 10% CCK-8 at 37°C for 4 h, and then, the optical density (OD) at a wavelength of 450 nm was measured with an absorbance microplate reader. All measurements were repeated at least three times.

Cell invasion was assessed with a Transwell chamber assay (Matrigel-coated membrane, Corning Costar Corp., Cambridge, MA, USA). According to the manufacturer’s instructions, a total of 1 × 10^5^ cells in each group (6 groups, as in the CCK-8 assay) were inoculated into the upper layer of an 8-μm Transwell chamber with 100–150 μl of serum-free DMEM/F12. The lower layer of the Transwell chamber was supplemented with DF12 medium containing 20% FBS. After 12 h of routine culture, the cells on the surface of the upper chamber were carefully removed with a cotton swab, and the cells that passed through the membrane were fixed with 4% polyformaldehyde for 30 min, followed by staining with crystal violet at 37°C for 1 h; the cells were then observed and counted using a CK40 inverted microscope (Olympus, Tokyo, Japan). The ratio of the number of cells that passed through to the total number of originally inoculated cells was defined as the invasion ratio.

### Evaluation of intracellular reporter gene expression

To determine the expression level of the reporter gene FTH1 in cells before and after malignant transformation, Western blotting (WB) was performed in each group of cells according to previously reported protocols [31]. Briefly, cells were lysed in radioimmunoprecipitation assay (RIPA) buffer (Beyotime) and then clarified by centrifugation at 14,000 rpm at 4°C for 15 min. The total protein concentration was determined by using the bicinchoninic acid (BCA; Beyotime) method. Then, 30 μg of protein was separated via 15% gradient SDS-polyacrylamide gel electrophoresis (SDS-PAGE; Beyotime) and transferred to polyvinylidene fluoride (PVDF) membranes (Millipore, Madrid, Spain); the membranes were blocked with 5% bovine serum albumin (BSA; Beyotime) in Tris-buffered saline containing Tween 20 (TBST) and then incubated with primary antibodies specific for FTH1 (rabbit anti-FTH1, 1:1,000; Abcam, Cambridge, MA, UK) or β-actin (rabbit anti-β-actin; Nanjing Zoonbino Biotechnology Co., Ltd., Nanjing, Jiangsu, China) overnight at 4°C. After washing three times with TBST, the membranes were incubated with secondary antibodies (anti-rabbit 1:5,000; anti-mouse 1:1,000; GenScript, Nanjing, Jiangsu, China) for 2 h and visualized using an enhanced chemiluminescence kit (Beyotime). FTH1 expression was quantified and normalized to β-actin expression using Quantity One 4.4 software (Bio-Rad, Hercules, CA, USA).

### Cellular MRI

Each group (six groups) of cells was cultured in medium supplemented with 0.5 mmol/L ferric ammonium citrate (FAC; Beyotime). After 72 h of culture, the cells were washed three times with PBS, trypsinized and collected by centrifuging at 1000 rpm for 5 min. A total of 4×10^7^ cells in each group were resuspended in 0.1 ml of PBS and carefully placed in 0.5-ml Eppendorf tubes for MRI. T2-weighted imaging (T2WI) was performed by using a clinical 3.0 T MRI scanner (Phillips, Eindhoven, Netherlands) with a knee coil; the parameters were as follows: time of repetition (TR)=2000 ms, time of echo (TE)=80 ms, field of view (FOV)=150 mm × 100 mm, imaging matrix = 380 × 311, and slice thickness=0.5 mm. A multiecho sequence was also performed with the following parameters: TR=2000 ms; TE=13~78 ms with a step size of 13 ms (six-point T2 mapping); and FOV, matrix, and slice thickness parameters that matched those used for T2WI. T2 maps were obtained on a postprocessing workstation, from which the T2 values were measured.

### Intracellular iron detection

Each group of cells was cultured in medium supplemented with 0.5 mmol/L FAC for 72 h, harvested and washed twice with PBS. For Prussian blue staining, cells were fixed in 4% paraformaldehyde (PFA) for 30 min and then stained with Prussian blue. Intracellular iron particles were visualized under a light microscope. For transmission electron microscopy (TEM) observation, cells were fixed in 2.5% glutaraldehyde cacodylate buffer at 4°C overnight, incubated in 1% OsO4 for 1 h, dehydrated in a graded ethanol series, and embedded in artificial EPON resin (Hexion, Shanghai, China). An H-7500 transmission electron microscope (Hitachi, Tokyo, Japan) was used to detect intracellular iron particles. For quantification of the intracellular iron content, 1×10^6^ cells per group were centrifuged and dried at 110°C overnight. The cells were then digested in 500 μl of a nitric acid-perchloric acid mixture (3:1) at 60°C for 3 h. The iron concentration was measured using an atomic absorption spectrophotometer (Huaguang HG-960 2A, Shenyang, China). Each sample was measured 3 times. The concentration values are presented in units of pg/cell.

### In vivo experiment

#### Cell xenografts

At least 2×10^6^ cells in each group (six groups, as in the CCK-8) were separately inoculated subcutaneously into nude mice (8 weeks, 18±3 g, male), and three mice were used in each group. The inoculation site in the nude mice was observed continuously at 1 week, 2 weeks, 3 weeks, and 4 weeks after cell inoculation. If a mass occurred, its diameter was recorded, and a mass growth curve was made. At 4 weeks, the mass was removed and observed under a microscope after hematoxylin and eosin (HE) staining. If no mass occurred, the observation period was extended to 8 weeks.

#### MRI of xenografts

To facilitate iron accumulation in cells for FTH1-based MRI detection in vivo, mice were fed 5 g/L FAC each day after cell inoculation. At 1 weeks, 2 weeks, 3 weeks, and 4 weeks after cell inoculation, MRI was performed after the mice were anesthetized by intraperitoneal injection of pentobarbital sodium at a dose of 30 mg/kg. A 3.0 T clinical MRI system with an animal coil was applied to perform T2WI and obtain a T2 map. The T2WI parameters were as follows: TR=2200 ms, TE=80 ms, FOV=180 mm × 150 mm, imaging matrix = 380 × 311, and slice thickness=1.2 mm. T2 mapping was performed by using a six-point multiecho T2WI sequence. The scanning parameters were the same as those used for cellular MRI, except the FOV was 180 mm × 150 mm, and the slice thickness was 1.2 mm. T2 values were measured from T2 color maps.

#### Iron detection in xenografts

After MRI, animals were sacrificed with an overdose of anesthesia, and the mass was removed, fixed with 4% PFA for more than 24 h, dehydrated with alcohol and xylol, embedded in paraffin, and cut into 4-μm sections. According to our previous protocols, Prussian blue staining was performed to confirm the existence of iron in the mass tissue. TEM was also used to observe iron particles in the mass tissue. In addition, the intratumoral iron content was quantified according to the protocols used for intracellular iron quantification and are presented in units of mg/g.

### Statistical analysis

Each experiment was repeated more than three times, and all quantitative data are expressed as the mean ± standard deviation. Statistical analyses were performed by using Statistical Package for the Social Sciences version 13.0 (SPSS Inc., Chicago, IL, USA). One-way analysis of variance (ANOVA) and Student’s *t* test were used for comparisons among groups or between two pairs. Differences were considered significant when the *P* value was less than 0.05.

## Results

### Identification of MSCs

**Fig. 2:**
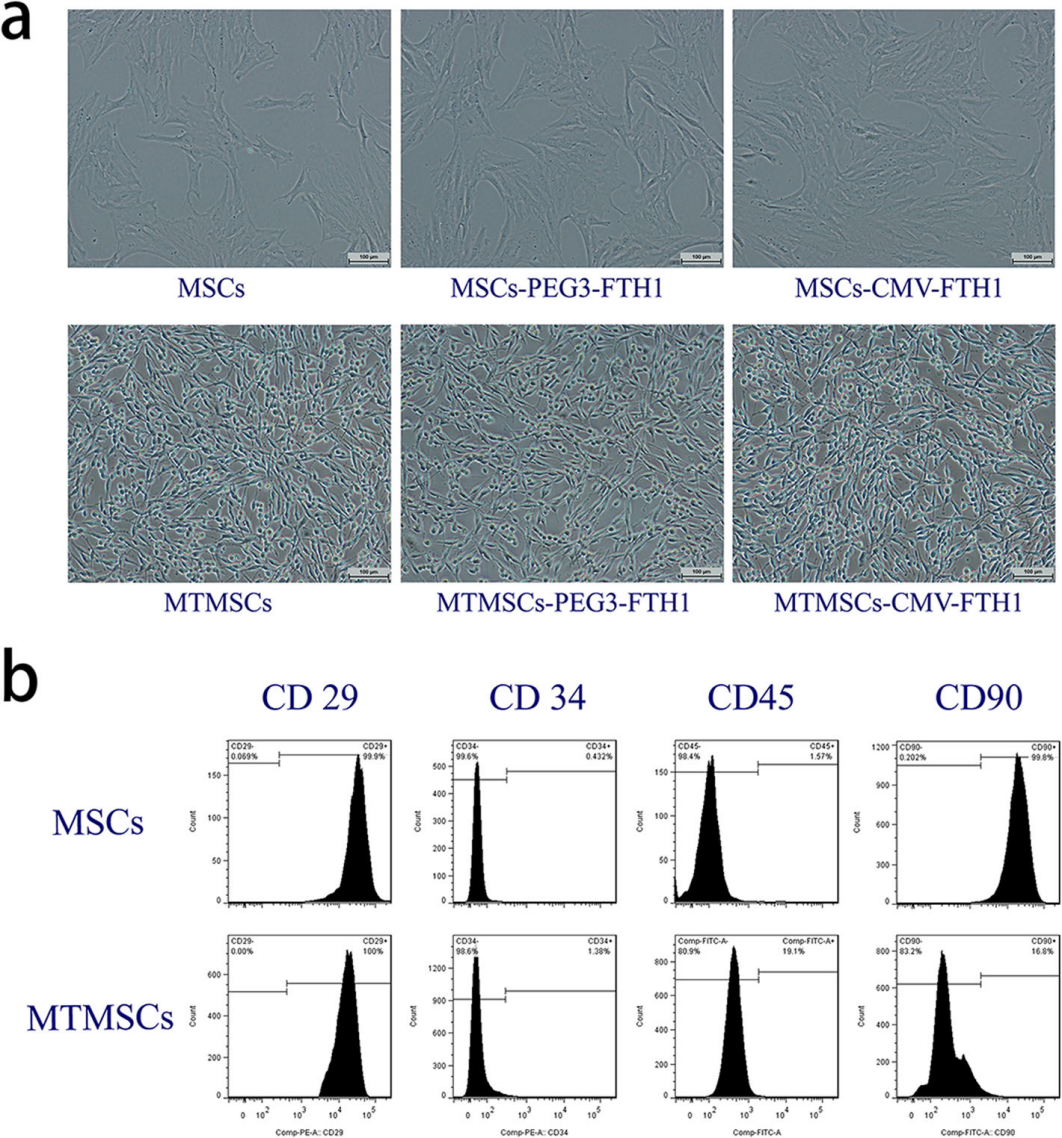
Cell morphology and surface antigens before and after malignant transformation. Compared with MSCs, cells that underwent malignant induction (MTMSCs) became small and demonstrated a spindle-like shape (**a**). Flow cytometric analysis showed that the expression of the MSC-specific cell surface antigen CD90 was significantly decreased (*P*<0.01), but that of CD45 was moderately increased after malignant transformation (**b**)

Microscopic observation demonstrated that the evaluated MSCs had a long spindle-shaped or fibroblast-like morphology, with a characteristic whirlpool arrangement (Fig. 2a). In addition, flow cytometry analysis showed that the CD29- and CD90-positive rates were 99.9% and 99.8%, respectively, while the CD34- and CD45-negative rates were 99.6% and 98.4%, respectively (Fig. 2b). These results confirmed that the cells used in this study were MSCs.

### Lentiviral construction and transduction

The FTH1 gene and PEG3 promoter in the target plasmid were verified to match the correct sequences. The observation of green fluorescence in 293T cells after infection suggested that viral packaging was successful. The best MOI for infection of MSCs with CMV-FTH1 and PEG3-FTH1 was determined to be 25. At the best MOI, the recombinant lentiviruses CMV-FTH1 and PEG3-FTH1 were transduced into MSCs, and MSCs-CMV-FTH1 and MSCs-PEG3-FTH1 were established successfully.

### Evaluation of malignant transformation

#### Morphology and surface antigens of cells

Compared with MSCs, MTMSCs displayed a small spindle-like shape, with a decreased cytoplasmic volume and an increased nucleocytoplasmic ratio, which are similar to the morphological features of tumor cells (Fig. 2a). Further flow cytometry analysis showed that the positive rates of the cellular surface antigens CD29 and CD90 for MTMSCs were 100% and 16.8%, respectively, while the CD34- and CD45-negative rates were 98.5% and 80.9%, respectively. The expression of CD90 was significantly decreased (*P*<0.01) and that of CD45 was moderately increased (*P*<0.05) after malignant transformation (Fig. 2b).

#### Cell proliferation and invasion

**Fig. 3:**
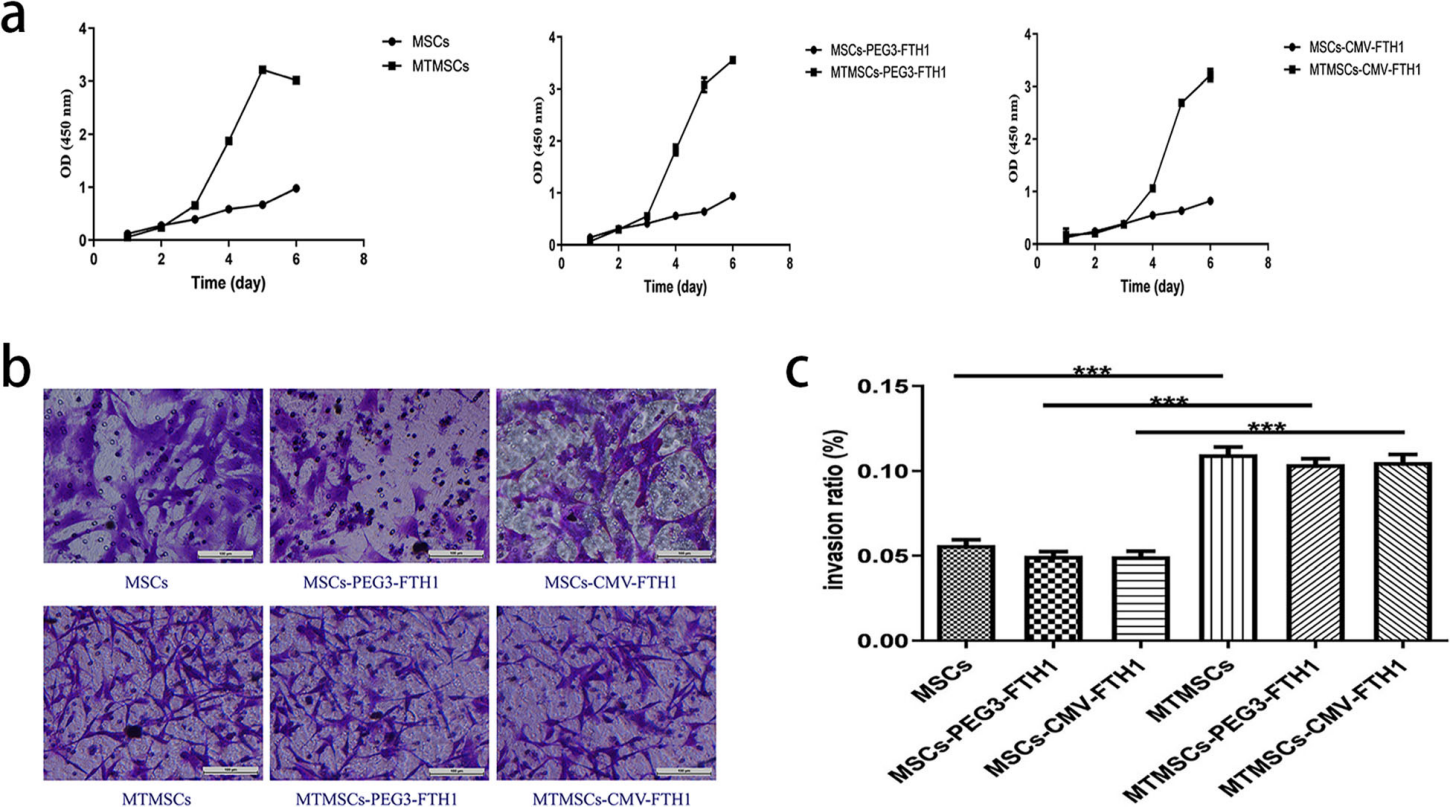
Cell proliferation and invasion before and after malignant transformation. Cell proliferation was significantly faster in the MTMSC, MTMSC-PEG3-FTH1, and MTMSC-CMV-FTH1 groups than in the MSC, MSC-PEG3-FTH1, and MSC-CMV-FTH1 groups, respectively, beginning on the 3rd day after the cells were seeded (**a**). The number of cells that migrated though the membrane was notably larger in the MTMSC, MTMSC-PEG3-FTH1, and MTMSC-CMV-FTH1 groups than in the MSC, MSC-PEG3-FTH1, and MSC-CMV-FTH1 groups, respectively (**b**). There were significant differences in the cell invasion ratio between the corresponding pre- and post-malignant transformation groups (**c**)

CCK-8 assay results showed that cell proliferation was significantly faster in the MTMSC, MTMSC-PEG3-FTH1, and MTMSC-CMV-FTH1 groups than in the MSC, MSC-PEG3-FTH1, and MSC-CMV-FTH1 groups, respectively, beginning on the 3rd day after cells were seeded (Fig. 3a). Furthermore, a Transwell chamber assay showed that the number of cells migrating though the membrane was notably larger in the MTMSC, MTMSC-PEG3-FTH1, and MTMSC-CMV-FTH1 groups than in the MSC, MSC-PEG3-FTH1, and MSC-CMV-FTH1 groups, respectively (Fig. 3b). There were significant differences in the cell invasion ratio between the corresponding pre- and post-malignant transformation groups (Fig. 3c).

### Cellular reporter gene expression

**Fig. 4:**
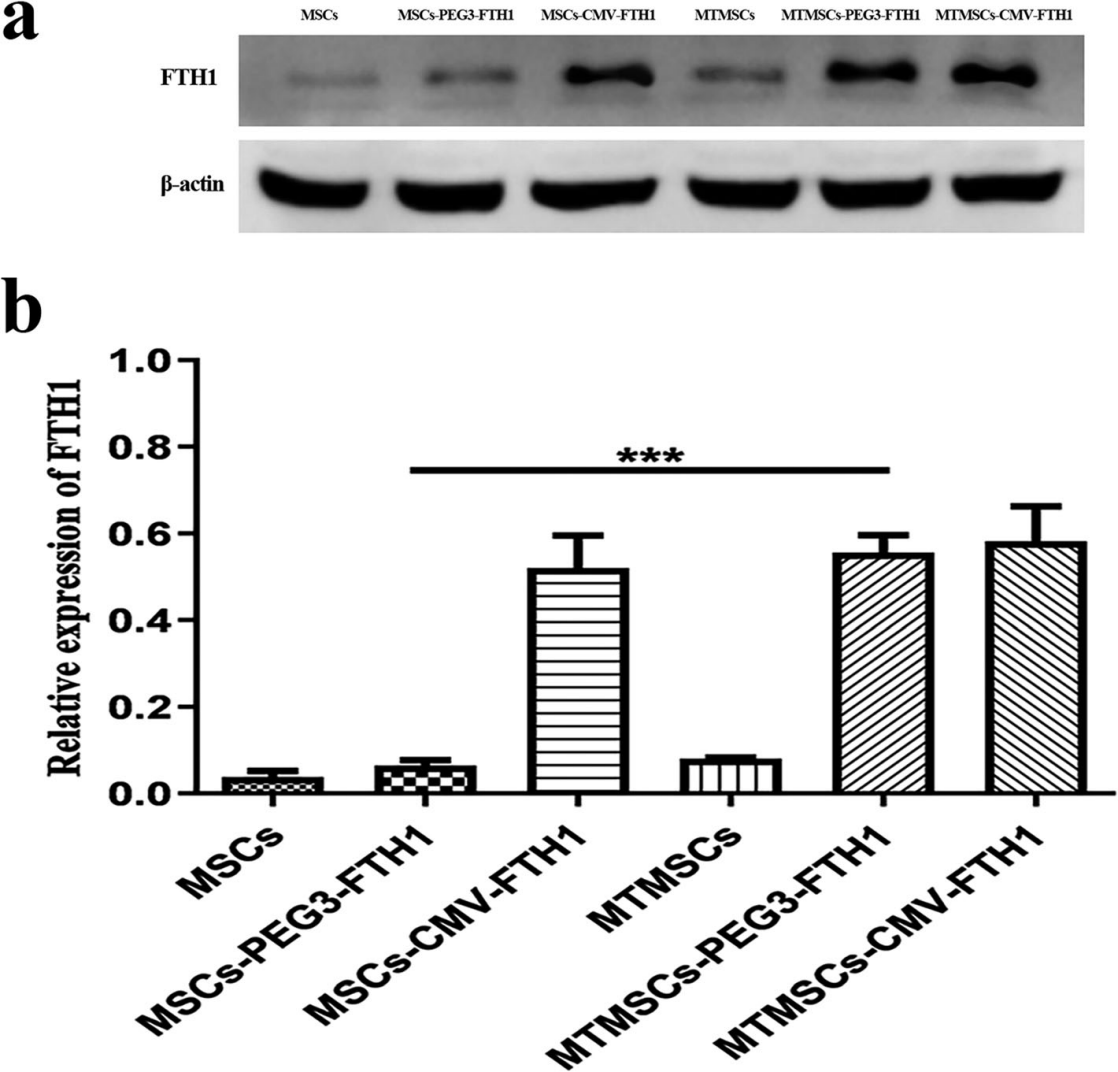
Cellular FTH1 expression before and after malignant transformation. Western blotting (**a**) and quantitative analysis (**b**) showed that the FTH1 expression in the MTMSCs-PEG3-FTH1 was significantly higher than that in the MSCs-PEG3-FTH1 (***P<0.001). However, there was no significant difference in FTH1 expression between the MTMSCs and MSCs or between the MTMSCs-CMV-FTH1 and MSCs-CMV-FTH1

WB results showed that FTH1 expression in MTMSCs-PEG3-FTH1 was significantly higher than that in MSCs-PEG3-FTH1. However, there was no significant difference in FTH1 expression between MTMSCs and MSCs or between MTMSCs-CMV-FTH1 and MSCs-CMV-FTH1 (Fig. 4a, b). These results showed that the promoter PEG3 could drive the expression of the downstream reporter gene specifically after malignant transformation, in contrast to the CMV promoter, which constitutively drove the reporter gene before and after malignant transformation.

### MRI signal contrast of cells

**Fig. 5:**
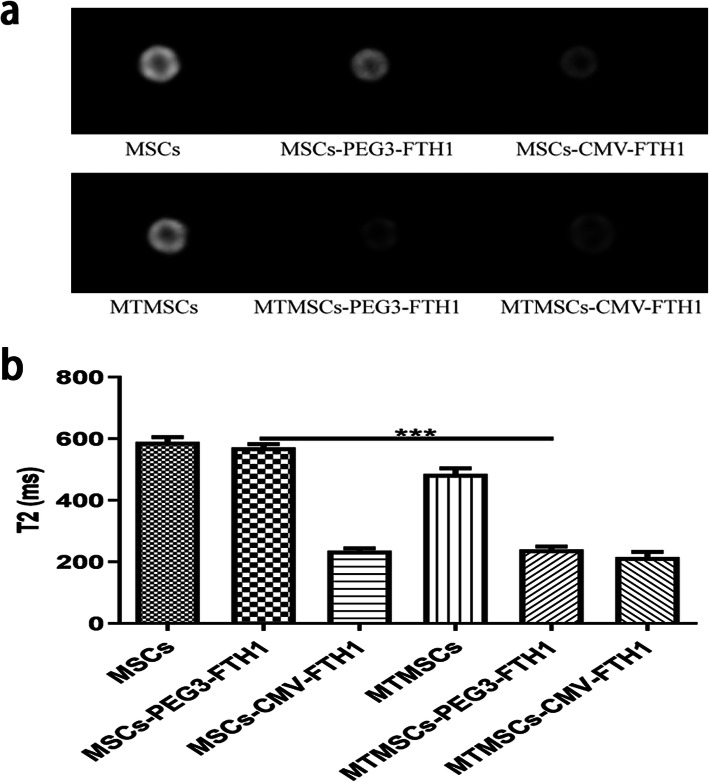
Cellular MRI before and after malignant transformation. The T2WI signal (**a**) and T2 value (**b**) were significantly decreased in the MTMSCs-PEG3-FTH1 compared with the MSCs-PEG3-FTH1 (***P<0.001), while no difference was found between the MTMSCs and MSCs or between the MTMSCs-CMV-FTH1 and MSCs-CMV-FTH1

MRI was performed to evaluate the signal contrast of MSCs before and after malignant transformation. As shown in Fig. 5, the cellular signal intensity on T2WI was markedly decreased in MTMSCs-PEG3-FTH1 compared with MSCs-PEG3-FTH1, and there was a significant difference in the T2 value between these two groups. Neither the T2WI signal intensity nor the T2 value was found to be different between MTMSCs and MSCs or between MTMSCs-CMV-FTH1 and MSCs-CMV-FTH1.

### Intracellular iron accumulation

**Fig. 6:**
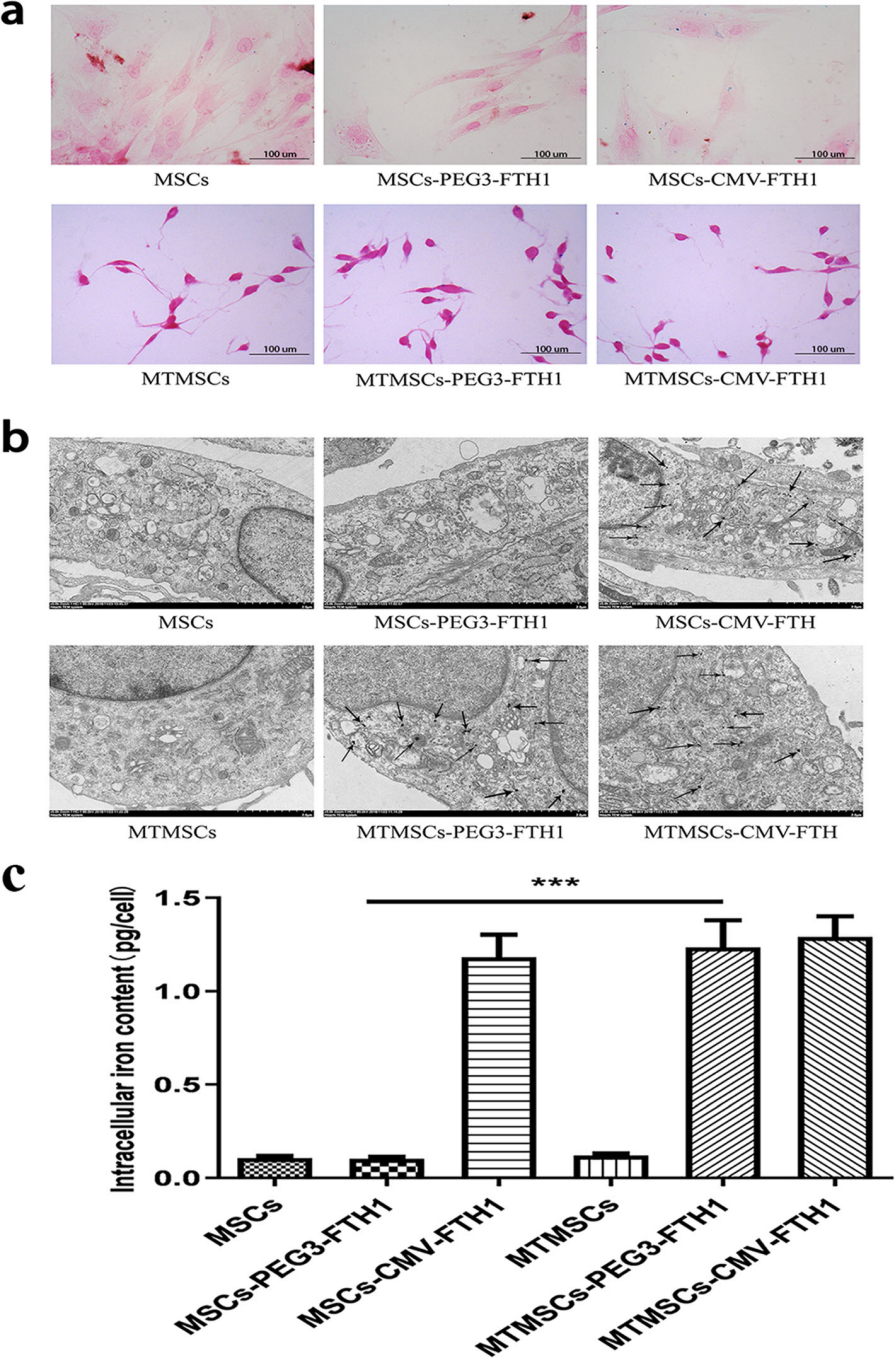
Intracellular iron observation. Prussian blue staining (**a**) and transmission electron microscopy (**b**) showed a large amount of intracytoplasmic iron particles in the MTMSCs-PEG3-FTH1, MSCs-CMV-FTH1, and MTMSCs-CMV-FTH1 but not in the MSCs, MTMSCs, or MSCs-PEG3-FTH1. Quantification of the intracellular iron content (**c**) showed that the iron content was significantly higher in MTMSCs-PEG3-FTH1 than in MSCs-PEG3-FTH1 (***P<0.001). There was no difference between the MTMSC-CMV-FTH1 and MSC-CMV-FTH1 groups or between the MTMSC and MSC groups

Prussian blue staining (Fig. 6a) and TEM (Fig. 6b) were used to evaluate the presence of intracellular iron particles. A large amount of intracytoplasmic iron particles was detected in the MTMSC-PEG3-FTH1, MSC-CMV-FTH1, and MTMSC-CMV-FTH1 groups but not in the MSC, MTMSC, or MSC-PEG3-FTH1 group. These results showed that the promoter PEG3 triggered FTH1 expression in a specific context, which caused iron collection in cells after malignant transformation. Quantification of the intracellular iron content (Fig. 6c) showed that the iron content in the MTMSCs-PEG3-FTH1 was 1.24 ± 0.18 pg/cell, significantly higher than that in MSCs-PEG3-FTH1 (0.10 ± 0.02 pg/cell) (*P*<0.001). There was no difference between the MTMSC-CMV-FTH1 (1.29 ± 0.17 pg/cell) and MSC-CMV-FTH1 (1.18 ± 0.20 pg/cell) groups or between the MTMSC (0.10 ± 0.02 pg/cell) and MSC (0.12 ± 0.02 pg/cell) groups.

### Tumorigenicity of xenografted cells

In the MSC, MSC-PEG3-FTH1, and MSC-CMV-FTH1 groups, no mass formation was found during the 8-week observation period after the cells were xenografted. In the MTMSC, MTMSC-PEG3-FTH1, and MTMSC-CMV-FTH1 groups; however, all mice were observed to grow a mass at the cell injection site. During the 1st week, the mass was palpable and similar to a granule. Then, the mass grew rapidly and could be detected by a clinical 3.0 T MRI system during the 2nd week.

### MRI of xenografts

**Fig. 7:**
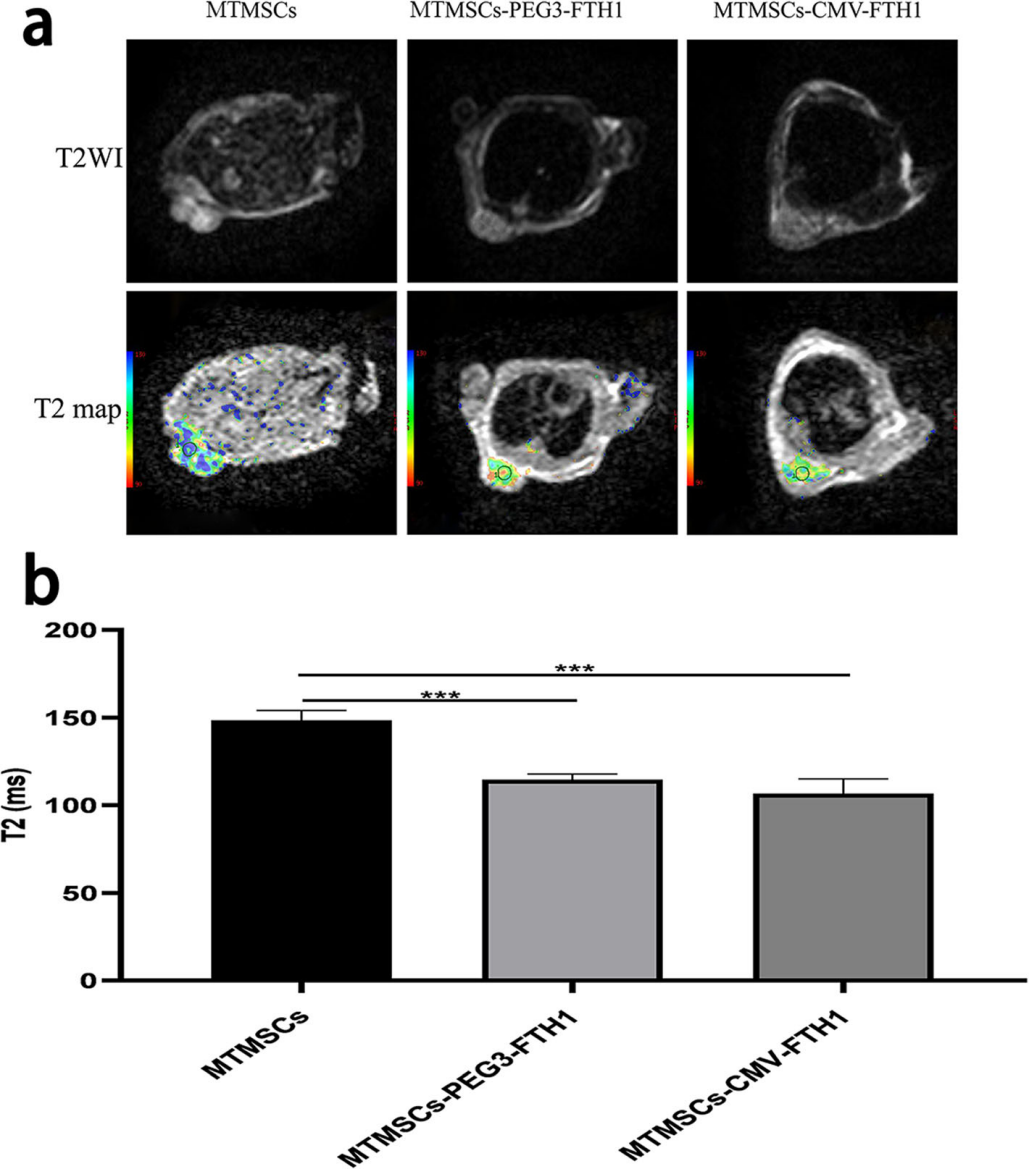
MRI of xenografts. The T2WI signal intensity (**a**) and T2 value (**b**) of masses were significantly decreased in the MTMSC-PEG3-FTH1 and MTMSC-CMV-FTH1 groups compared with the MTMSC group. There was no significant difference in the T2 value between the MTMSC-PEG3-FTH1 group and the MTMSC-CMV-FTH1 group

As shown in Fig. 7, the T2WI signal intensity and T2 value of masses were both significantly decreased in the MTMSC-PEG3-FTH1 and MTMSC-CMV-FTH1 groups compared with the MTMSC group. There was no significant difference in the T2 value between the MTMSC-PEG3-FTH1 group and the MTMSC-CMV-FTH1 group.

### Pathological examination of xenografts

**Fig. 8:**
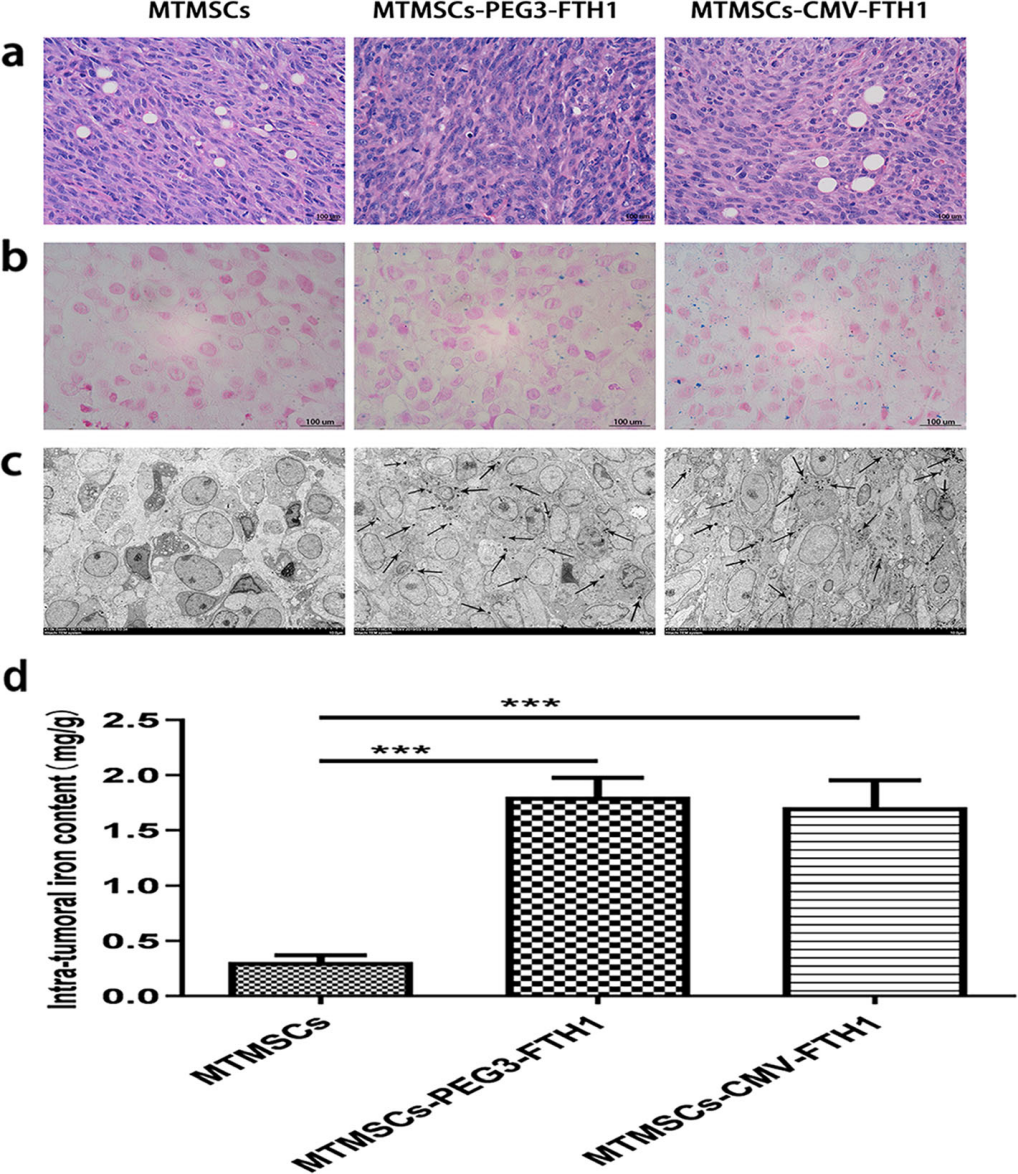
Pathological characteristics of xenografts. HE staining showed that the cells in the mass were arranged close together and that the cytoplasmic ratio was relatively large (**a**), which conformed to the morphology of tumor tissue. Prussian blue staining (**b**) and TEM (**c**) showed a large amount of iron particles in the mass tissue in the MTMSC-PEG3-FTH1 and MTMSC-CMV-FTH1 groups but not in the MTMSC group. The intratumoral iron content (**d**) was not significantly different between the MTMSC-PEG3-FTH1 and MTMSC-CMV-FTH1 groups but was significantly higher in both of these groups than in the MTMSC group (***P<0.001)

HE staining of masses showed that the cells were arranged closely and that the cytoplasmic ratio was relatively large (Fig. 8a), which conformed to the morphological characteristics of tumor tissue.

Both Prussian blue staining (Fig. 8b) and TEM (Fig. 8c) showed massive iron particles distributed in the mass tissue in the MTMSC-PEG3-FTH1 and MTMSC-CMV-FTH1 groups but not in the MTMSC group. The intratumoral iron content (Fig. 8d) was not significantly different between the MTMSC-PEG3-FTH1 group (1.81 ± 0.19 mg/g) and the MTMSC-CMV-FTH1 group (1.72 ± 0.23 mg/g). However, the intratumoral iron content in both of these groups was significantly higher than that in the MTMSC group (0.30 ± 0.04 mg/g). These results showed that iron accumulation resulting from expression of the reporter gene FTH1 contributed to the T2WI signal decrease in cell xenografts. FTH1 expression driven by the promoter PEG3 could cause iron collection equivalent to that driven by the CMV promoter.

## Discussion

The goal of this study was to develop an MRI monitoring system that could sensitively detect the potential malignant transformation of stem cells as early as possible. For such a purpose, two conditions should be satisfied. One is that the MRI reporter gene should be incorporated into cells in advance. The other is that the reporter gene should be expressed in a tumor-specific manner. Previous studies have revealed that FTH1, which can be transduced into stem cells and cause intracellular iron accumulation allowing for MRI signal change via overexpression, can be used as an ideal genetic MRI reporter for longitudinal monitoring of stem cells [18–20, 32]. However, this strategy cannot be used to detect the occurrence of malignant stem cell transformation because the reporter gene is continuously expressed regardless of how the cells transform. To solve this problem, we modified the reporter gene FTH1 by adding the tumor-specific promoter PEG3 upstream and successfully transferred the modified reporter gene into stem cells with a recombinant lentivirus. When stem cells were induced to undergo malignant transformation, the PEG3 promoter was activated and automatically triggered the expression of the reporter gene FTH1, which allowed for MRI detection. This study realized the automatic switching function of the MRI reporter gene, that is, the reporter gene remained silent in normal stem cells but turned on when stem cells transformed into tumor cells. This MRI tracing system could be applied to detect unexpected tumorigenicity in stem cell-based therapeutic strategies and could even be extended to monitor the elimination of malignantly transformed cells in combination with a therapeutic gene.

PEG3, a rodent-derived gene, was identified by using the subtraction hybridization method while searching for genes involved in malignant transformation and tumor progression [22]. Because of its tumor-specific nature and activity in most human tumors, PEG3 has been applied as a potential tumor-specific promoter for diagnostic and therapeutic purposes. Studies have shown that the PEG3 promoter can drive the expression of the luciferase gene in tumor cells, allowing detection with bioluminescence imaging, which indicates that the PEG3 promoter has the ability to drive specific expression of downstream reporter genes in cancer cells or tissues [33]. In previous studies, many tumor-specific promoters, such as the mucin-1 promoter for breast cancer [34], the prostate-specific antigen (PSA) promoter for prostate cancer [35], and the mesothelin promoter for ovarian cancer [36], have been investigated for use in the detection or treatment of primary tumors and their metastatic lesions. In contrast to those promoters, the PEG3 promoter has been broadly tested and found to be active in a variety of tumors, including breast, brain, prostate, and pancreatic cancers [22]. Although some other promoters, such as hTERT, hiwi, and survivin, have been reported to be expressed across a relatively broad spectrum of cancers [37–39], these promoters are also active in stem cells, in which they are considered to maintain the self-renewal and proliferative features [40–42]. Therefore, these promoters are not suitable for this study design, which required differential expression of the promoter before and after malignant stem cell transformation. In addition to its specificity for tumors, another advantage of the PEG3 promoter is its high activity in tumor cells. In comparison with hTERT and survivin, PEG3 is directly responsive to transcription factors unique to tumor cells rather than requiring reliance on the transcriptional level of a marker gene. The high activity of the PEG3 promoter in human cancer cells can be attributed to the cooperation of the two transcription factors AP-1 and PEA-3 [23]. In a recent study that investigated the targeted therapeutic effect of the apoptosis-inducing gene sTRAIL on teratocarcinoma, the cancer cell-specific promoter PEG3 or hTERT was applied to drive therapeutic gene expression, and the results demonstrated that both promoters could drive target gene expression and enable effective gene therapy against teratocarcinoma but that the promoter activity of the PEG3 promoter was more robust than that of the hTERT promoter [25]. In this study, we compared PEG3 with the CMV promoter in the context of triggering a reporter gene in malignantly transformed stem cells. The WB results showed that the expression of FTH1 in MTMSC-PEG3-FTH1 cells was significantly upregulated compared with that in MSC-PEG3-FTH1 cells, indicating that the PEG3 promoter activates and drives FTH1 expression during the transformation of MSCs-PEG3-FTH1 into MTMSCs-PEG3-FTH1, with a driving capability as strong as that of the CMV promoter.

MSCs have the potential risk of undergoing malignant transformation, which may impede the application of stem cell-based therapeutic strategies. It has been reported that MSCs can be recruited to tumors and participate in the formation of the tumor stroma [43–47]. Recent studies have shown that MSCs can undergo malignant transformation when they are cultured for a long period of time or in a tumor-like environment, although the underlying mechanism remains unknown [10, 30, 48, 49]. In this study, we induced MSCs to undergo malignant transformation by indirectly coculturing them with C6 glioma cells. After 2 weeks of induction, the cells showed tumor cell features in terms of morphology, proliferation, migration, and invasion. In addition, flow cytometry revealed that the expression of CD90, a cell-surface marker of MSCs, was significantly decreased after malignant induction. This observation was in line with the results of a study in which MSCs were induced to spontaneously undergo malignant transformation by long-term culture [10]. To further evaluate the in vivo tumorigenicity of MTMSCs, these cells were injected subcutaneously into immunodeficient mice. After a short period of time, all the induced cell grafts developed into masses at the injection site. The above evidence suggested that the MSCs had undergone malignant transformation. Using this MSC malignant transformation model, we investigated the difference in the expression of the reporter gene FTH1 driven by the promoter PEG3 before and after malignant transformation. The results showed that PEG3 could trigger reporter gene expression only after cells underwent malignant induction, not before malignant induction. This tumor-specific expression of the reporter gene could potentially be applied to monitor the malignant transformation of stem cells, which are usually used as therapeutic cells targeting the tumor environment in vivo.

Unfortunately, we did not detect malignant transformation of stem cells in vivo during 8 weeks of observation in the MSC xenograft group, which could be due to the variability in MSC tumorigenesis. Although induced stem cells (MTMSCs) implanted into nude mice successfully formed masses, in which the ability of the triggered reporter gene expression to drive iron collection and allow subsequent MRI detection was confirmed, these cells could not truly mimic the natural tumor transformation process that occurs after stem cell transplantation. This was one of the main limitations of the study. In the future, attempts should be made to use stem cells derived from different sources to establish a more suitable tumorigenicity model for verifying the feasibility of using the PEG3 promoter-triggered genetic reporter imaging system in vivo to monitor the malignant transformation of stem cells after transplantation.

In conclusion, we developed a novel reporter gene-based MRI monitoring system for the detection of the malignant transformation of stem cells by modifying the genetic reporter FTH1 to be under the control of the tumor-specific promoter PEG3. For the imaging system, the reporter gene remained silent in stem cells, while its expression was triggered by the promoter when the stem cells underwent malignant transformation. This enabled the reporter gene to have a “switching on” function and allowed MRI to sensitively detect the occurrence of stem cell tumorigenicity. The tumor-specific MRI monitoring system could potentially enhance the safety of stem cell-based therapeutic strategies by detecting malignantly transformed stem cells as early as possible or even by eliminating transformed cells in combination with some therapeutic genes.

## Data Availability

The datasets used and/or analyzed during the current study are available from the corresponding author on reasonable request.

## Abbreviations

MSC: Mesenchymal stem cell
MRI: Magnetic resonance imaging
FTH1: Ferritin heavy chain
PEG3: Progression elevated gene-3
LV: Lentiviral vector
MTMSC: Malignantly transformed mesenchymal stem cell
DMEM: Dulbecco’s modified Eagle’s medium
FBS: Fetal bovine serum
PBS: Phosphate-buffered saline
PCR: Polymerase chain reaction
MOI: Multiplicity of infection
CCK-8: Cell Counting Kit-8
OD: Optical density
WB: Western blotting
RIPA: Radioimmunoprecipitation assay
TBST: Tris-buffered saline containing Tween 20
FAC: Ferric ammonium citrate
T2WI: T2-weighted imaging
TR: Time of repetition
TE: Time of echo
FOV: Field of view
TEM: Transmission electron microscopy
HE: Hematoxylin and eosin

## References

[1] Fathi F, Rezabakhsh A, Rahbarghazi R, Rashidi MR (2017). Early-stage detection of VE-cadherin during endothelial differentiation of human mesenchymal stem cells using SPR biosensor. Biosens Bioelectron..

[2] Glenn JD, Whartenby KA (2014). Mesenchymal stem cells: emerging mechanisms of immunomodulation and therapy. World J Stem Cells..

[3] Mohammadipoor A, Antebi B, Batchinsky AI, Cancio LC (2018). Therapeutic potential of products derived from mesenchymal stem/stromal cells in pulmonary disease. Respir Res..

[4] Shende P, Gupta H, Gaud RS (2018). Cytotherapy using stromal cells: current and advance multi-treatment approaches. Biomed Pharmacother..

[5] Ullah I, Subbarao RB, Rho GJ (2015). Human mesenchymal stem cells - current trends and future prospective. Biosci Rep..

[6] Lye KL, Nordin N, Vidyadaran S, Thilakavathy K (2016). Mesenchymal stem cells: from stem cells to sarcomas. Cell Biol Int..

[7] Miura M, Miura Y, Padilla-Nash HM, Molinolo AA, Fu B, Patel V, Seo BM, Sonoyama W, Zheng JJ, Baker CC, Chen W, Ried T, Shi S (2006). Accumulated chromosomal instability in murine bone marrow mesenchymal stem cells leads to malignant transformation. Stem Cells..

[8] Nurkovic J, Dolicanin Z, Mustafic F, Mujanovic R, Memic M, Grbovic V, Skevin AJ, Nurkovic S (2016). Mesenchymal stem cells in regenerative rehabilitation. J Phys Ther Sci..

[9] Reya T, Morrison SJ, Clarke MF, Weissman IL (2001). Stem cells, cancer, and cancer stem cells. Nature..

[10] Røsland GV, Svendsen A, Torsvik A, Sobala E, McCormack E, Immervoll H, Mysliwietz J, Tonn JC, Goldbrunner R, Lønning PE, Bjerkvig R, Schichor C (2009). Long-term cultures of bone marrow–derived human mesenchymal stem cells frequently undergo spontaneous malignant transformation. Cancer Res..

[11] Mathiasen AB, Kastrup J (2013). Non-invasive in-vivo imaging of stem cells after transplantation in cardiovascular tissue. Theranostics..

[12] Struys T, Ketkar-Atre A, Gervois P, Leten C, Hilkens P, Martens W, Bronckaers A, Dresselaers T, Politis C, Lambrichts I, Himmelreich U (2013). Magnetic resonance imaging of human dental pulp stem cells in vitro and in vivo. Cell Transplant..

[13] Fink J, Andersson-Rolf A, Koo BK (2015). Adult stem cell lineage tracing and deep tissue imaging. BMB Rep..

[14] Zhang J, Yuan Y, Han Z, Li Y, van Zijl PCM, Yang X, Bulte JWM, Liu G (2019). Detecting acid phosphatase enzymatic activity with phenol as a chemical exchange saturation transfer magnetic resonance imaging contrast agent (PhenolCEST MRI). Biosens Bioelectron..

[15] Brewer KD, Spitler R, Lee KR, Chan AC, Barrozo JC, Wakeel A, Foote CS, Machtaler S, Rioux J, Willmann JK, Chakraborty P, Rice BW, Contag CH, Bell CB, Rutt BK (2018). Characterization of magneto-endosymbionts as MRI cell labeling and tracking agents. Mol Imaging Biol..

[16] Qin Y, Zhuo L, Cai J, He X, Liu B, Feng C (2017). In vivo monitoring of magnetically labeled mesenchymal stem cells homing to rabbit hepatic VX2 tumors using magnetic resonance imaging. Mol Med Rep..

[17] Skelton RJP, Khoja S, Almeida S, Rapacchi S, Han F, Engel J, Zhao P, Hu P, Stanley EG, Elefanty AG, Kwon M, Elliott DA, Ardehali R (2016). Magnetic resonance imaging of iron oxide-labeled human embryonic stem cell-derived cardiac progenitors. Stem Cells Transl Med..

[18] He X, Cai J, Li H, Liu B, Qin Y, Zhong Y, Wang L, Liao Y (2016). In Vivo magnetic resonance imaging of xenografted tumors using FTH1 reporter gene expression controlled by a tet-on switch. Oncotarget..

[19] Kim HS, Woo J, Choi Y, Hwang EH, Choi SK, Cho KW (2014). Noninvasive MRI and multilineage differentiation capability of ferritin-transduced human mesenchymal stem cells. NMR Biomed..

[20] Mu T, Qin Y, Liu B, He X, Liao Y, Sun J (2018). In vitro neural differentiation of bone marrow mesenchymal stem cells carrying the FTH1 reporter gene and detection with MRI. BioMed Res Int..

[21] Yahyapour R, Farhood B, Graily G, Rezaeyan A, Rezapoor S, Abdollahi H, Cheki M, Amini P, Fallah H, Najafi M, Motevaseli E (2018). Stem cell tracing through MR molecular imaging. Tissue Eng Regen Med..

[22] Su ZZ, Shi Y, Fisher PB (1997). Subtraction hybridization identifies a transformation progression-associated gene PEG-3 with sequence homology to a growth arrest and DNA damage-inducible gene. Proc Natl Acad Sci U S A..

[23] Su ZZ, Shi Y, Fisher PB (2000). Cooperation between AP1 and PEA3 sites within the progression elevated gene-3 (PEG-3) promoter regulate basal and differential expression of PEG-3 during progression of the oncogenic phenotype in transformed rat embryo cells. Oncogene..

[24] Emdad L, Sarkar D, Su ZZ, Boukerche H, Bar-Eli M, Fisher PB (2004). Progression elevated gene-3 (PEG-3) induces pleiotropic effects on tumor progression: modulation of genomic stability and invasion. J Cell Physiol..

[25] Jiang X, Du LL, Yang S, Chen LS, Lu GX (2009). Suppression of teratocarcinoma growth by soluble TRAIL gene driven by the progression-elevated gene-3 promoter. Cancer Biol Ther..

[26] Minn I, Bar-Shir A, Yarlagadda K, Bulte JWM, Fisher PB, Wang H, Gilad AA, Pomper MG (2015). Tumor-specific expression and detection of a CEST reporter gene. Magn Reson Med..

[27] Sarkar D, Lebedeva IV, Su ZZ, Park ES, Chatman L, Vozhilla N, Dent P, Curiel DT, Fisher PB (2007). Eradication of therapy-resistant human prostate tumors using a cancer terminator virus. Cancer Res..

[28] Su ZZ, Emdad L, Sarkar D, Randolph A, Valerie K, Yacoub A, Dent P, Fisher PB (2005). Potential molecular mechanism for rodent tumorigenesis: mutational generation of Progression Elevated Gene-3 (PEG-3). Oncogene..

[29] Cai J, Zhang X, Wang X, Li C, Liu G (2008). In vivo MR imaging of magnetically labeled mesenchymal stem cells transplanted into rat liver through hepatic arterial injection. Contrast Media Mol Imaging..

[30] Tan B, Shen L, Yang K, Huang D, Li X, Li Y, Zhao L, Chen J, Yi Q, Xu H, Tian J, Zhu J (2018). C6 glioma-conditioned medium induces malignant transformation of mesenchymal stem cells: possible role of S100B/RAGE pathway. Biochem Biophys Res Commun..

[31] He X, Cai J, Liu B, Zhong Y, Qin Y (2015). Cellular magnetic resonance imaging contrast generated by the ferritin heavy chain genetic reporter under the control of a Tet-On switch. Stem Cell Res Ther..

[32] Naumova AV, Reinecke H, Yarnykh V, Deem J, Yuan C, Murry CE (2010). Ferritin overexpression for noninvasive magnetic resonance imaging–based tracking of stem cells transplanted into the heart. Mol Imaging..

[33] Bhang HEC, Gabrielson KL, Laterra J, Fisher PB, Pomper MG (2011). Tumor-specific imaging through progression elevated gene-3 promoter-driven gene expression. Nat Med..

[34] Huyn ST, Burton JB, Sato M, Carey M, Gambhir SS, Wu L (2009). A potent, imaging adenoviral vector driven by the cancer-selective mucin-1 promoter that targets breast cancer metastasis. Clin Cancer Res..

[35] Lu Y, Zhang Y, Chang G, Zhang J (2013). Comparison of prostate-specific promoters and the use of PSP-driven virotherapy for prostate cancer. BioMed Res Int..

[36] Breidenbach M, Rein DT, Everts M, Glasgow JN, Wang M, Passineau MJ (2004). Mesothelin-mediated targeting of adenoviral vectors for ovarian cancer gene therapy. Gene Ther..

[37] Mizukoshi E, Kaneko S (2019). Telomerase-targeted cancer immunotherapy. Int J Mol Sci..

[38] Rodel F, Sprenger T, Kaina B, Liersch T, Rodel C, Fulda S, Hehlgans S (2012). Survivin as a prognostic/predictive marker and molecular target in cancer therapy. Curr Med Chem..

[39] Siddiqi S, Terry M, Matushansky I (2012). Hiwi mediated tumorigenesis is associated with DNA hypermethylation. PLoS One..

[40] Taubert H, Kappler M, Bache M, Bartel F, Köhler T, Lautenschläger C, Blümke K, Würl P, Schmidt H, Meye A, Hauptmann S (2005). Elevated expression of survivin-splice variants predicts a poor outcome for soft-tissue sarcomas patients. Oncogene..

[41] Taubert H, Greither T, Kaushal D, Würl P, Bache M, Bartel F (2006). Expression of the stem cell self-renewal gene Hiwi and risk of tumour-related death in patients with soft-tissue sarcoma. Oncogene..

[42] Taubert H, Würl P, Greither T, Kappler M, Bache M, Bartel F, Kehlen A, Lautenschläger C, Harris LC, Kaushal D, Füssel S, Meye A, Böhnke A, Schmidt H, Holzhausen HJ, Hauptmann S (2007). Stem cell-associated genes are extremely poor prognostic factors for soft-tissue sarcoma patients. Oncogene..

[43] Chen J, Ji T, Wu D, Jiang S, Zhao J, Lin H, Cai X (2019). Human mesenchymal stem cells promote tumor growth via MAPK pathway and metastasis by epithelial mesenchymal transition and integrin α5 in hepatocellular carcinoma. Cell Death Dis..

[44] Doucette T, Rao G, Yang Y, Gumin J, Shinojima N, Bekele BN, Qiao W, Zhang W, Lang FF (2011). Mesenchymal stem cells display tumor-specific tropism in an RCAS/Ntv-a glioma model. Neoplasia..

[45] He Q, Zou X, Duan D, Liu Y, Xu Q (2015). Malignant transformation of bone marrow stromal cells induced by the brain glioma niche in rats. Mol Cell Biochem..

[46] Pavon LF, Sibov TT, de Souza AV, da Cruz EF, Malheiros SMF, Cabral FR, de Souza JG, Boufleur P, de Oliveira DM, de Toledo SRC, Marti LC, Malheiros JM, Paiva FF, Tannús A, de Oliveira SM, Chudzinski-Tavassi AM, de Paiva Neto MA, Cavalheiro S (2018). Tropism of mesenchymal stem cell toward CD133(+) stem cell of glioblastoma in vitro and promote tumor proliferation in vivo. Stem Cell Res Ther..

[47] Pietrovito L, Leo A, Gori V, Lulli M, Parri M, Becherucci V, Piccini L, Bambi F, Taddei ML, Chiarugi P (2018). Bone marrow-derived mesenchymal stem cells promote invasiveness and transendothelial migration of osteosarcoma cells via a mesenchymal to amoeboid transition. Mol Oncol..

[48] Ji R, Zhang X, Qian H, Gu H, Sun Z, Mao F (2017). miR-374 mediates the malignant transformation of gastric cancer-associated mesenchymal stem cells in an experimental rat model. Oncol Rep.

[49] Zhang YM, Zhang ZM, Guan QL, Liu YQ, Wu ZW, Li JT, Su Y, Yan CL, Luo YL, Qin J, Wang Q, Xie XD (2017). Co-culture with lung cancer A549 cells promotes the proliferation and migration of mesenchymal stem cells derived from bone marrow. Exp Ther Med..

